# Cardiac Allograft Vasculopathy: A Donor or Recipient Induced Pathology?

**DOI:** 10.1007/s12265-015-9612-x

**Published:** 2015-02-05

**Authors:** Patricia van den Hoogen, Manon M. H. Huibers, Joost P. G. Sluijter, Roel A. de Weger

**Affiliations:** 1 Department of Cardiology, Experimental Cardiology laboratory, University Medical Centre Utrecht, Utrecht, The Netherlands; 2 Department of Pathology, University Medical Center Utrecht, Room H04.312 Heidelberglaan 100, PO Box 85500, 3508 GA Utrecht, The Netherlands

**Keywords:** Heart transplantation, Cardiac allograft vasculopathy, Donor cells, Recipient cells, Immune response, Fibrosis, Mismatch, Circulating cells

## Abstract

Cardiac allograft vasculopathy (CAV) is one of the main causes of late-stage heart failure after heart transplantation. CAV is characterized by concentric luminal narrowing of the coronary arteries, but the exact pathogenesis of CAV is still not unraveled. Many researchers show evidence of an allogeneic immune response of the recipient, whereas others show contrasting results in which donor-derived cells induce an immune response against the graft. In addition, fibrosis of the neo-intima can be induced by recipient-derived circulating cells or donor-derived cells. In this review, both donor and recipient sides of the story are described to obtain better insight in the pathogenesis of CAV. Dual outcomes were found regarding the contribution of donor and recipient cells in the initiation of the immune response and the development of fibrosis during CAV. Future research could focus more on the potential synergistic interaction of donor and recipient cells leading to CAV.

## Introduction

Cardiac transplantation is often successfully applied in the treatment of end-stage heart failure [1]. Since 1982, more than 110,000 heart transplantations have been performed globally and these numbers are still rising [2]. Over the years, early survival rates of recipients, which received a heart transplantation, have significantly improved [2]. In the first months after transplantation, acute rejection of the transplanted heart can occur [3]. Much progress has been made in controlling this acute rejection phase, resulting in increased early survival rates [2]. However, chronic rejection is one of the major issues that affects long-term survival of heart transplant recipients [4].

One of the main causes of chronic rejection is cardiac allograft vasculopathy (CAV) [1–5]. CAV is an accelerated form of coronary artery disease [6] and affects both males and females [3]. The mechanism by which CAV develops is not fully elucidated, but it is estimated that 50 % of heart transplantation recipients are developing CAV within 5 years after transplantation [7]. Hence, CAV is responsible for 10–15 % of cardiac deaths after transplantation [8]. CAV affects the vasculature of the transplanted heart, resulting in congestive heart failure, arrhythmias, myocardial infarction, or sudden cardiac death [1, 9]. Both immunologic factors and non-immunologic factors, such as age, gender, and brain injury, are involved in the development of CAV, although immunologic factors have shown to be the most important players [5, 10].

**Fig. 1:**
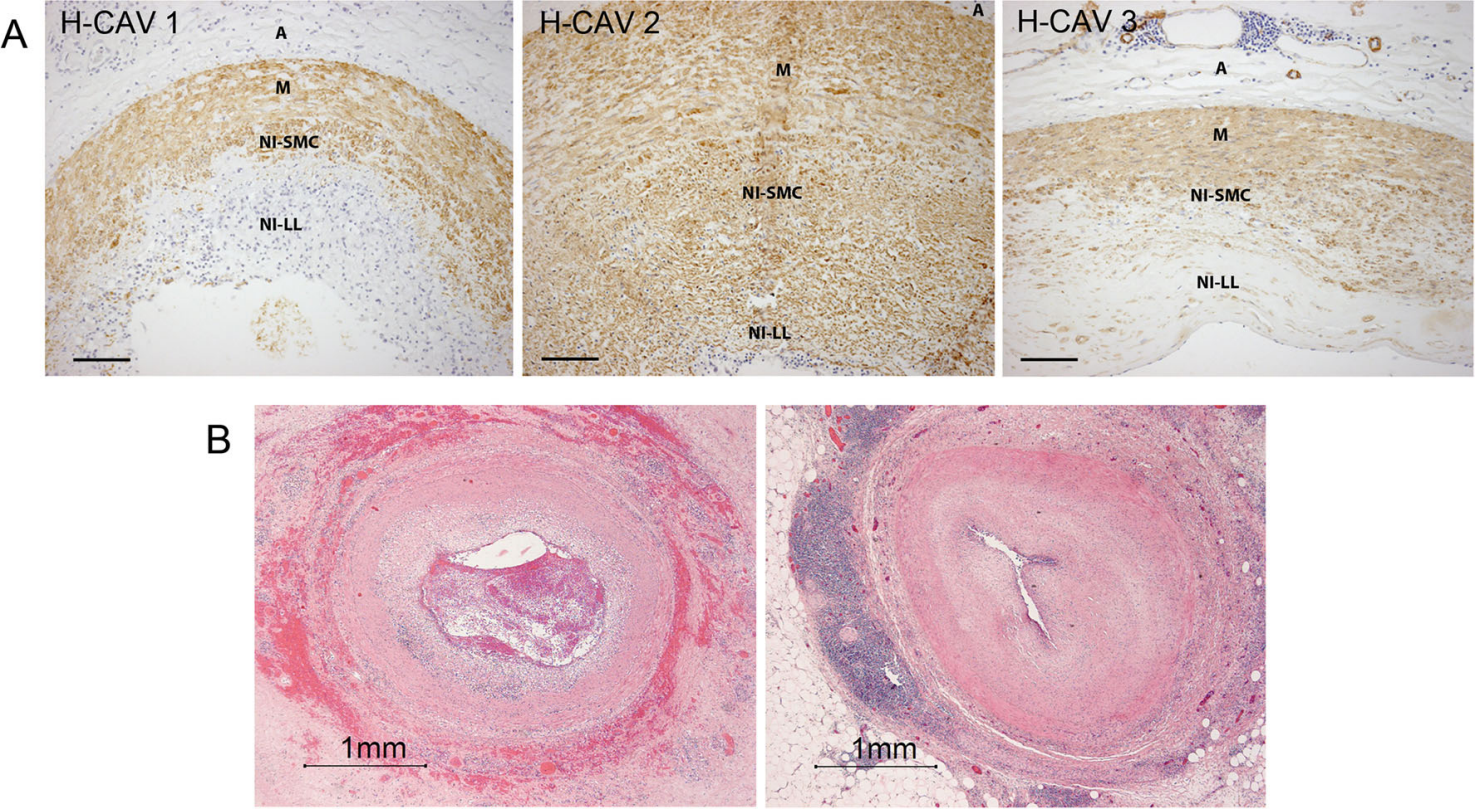
Microscopic pictures of the three histopathological phenotypes of CAV in the coronary artery of heart transplantation recipients. **a** H-CAV 1 lesion, which shows infiltration of lymphocytes in the neo-intima layer; H-CAV 2 lesion, showing infiltration of lymphocytes together with infiltration of smooth muscle cells and formation of connective tissue; H-CAV 3 lesion, which shows a large fibrotic intimal lesion without inflammatory infiltrate (αSMA staining, magnification ×100, line indicates 100 μm). **b** Microscopic pictures of occluded coronary arteries by a thrombus or fibrotic tissue, respectively (HE staining, magnification ×20, line indicates 1 mm)

CAV is characterized by diffuse intimal thickening leading to progressive narrowing of the coronary arteries [5, 11]. There are different types of lesions in CAV patients, including intimal hyperplasia, atherosclerotic lesions, and vasculitis [3]. Within the lesions of intimal hyperplasia, three histopathological phenotypes of CAV can be observed: 1) loose connective tissue with inflammatory cells, 2) lesions with smooth muscle cells, and 3) fibrotic lesions (Fig. 1) [12]. Most commonly seen characteristic in CAV is fibromuscular hyperplasia of the intima, which also distinguishes CAV from atherosclerosis [8]. Ultimately, progressive narrowing of the coronary artery results in critical stenosis and ischemia of the graft [3].

The exact mechanism in which CAV is induced after heart transplantation is not elucidated, but it is known that both donor and recipient cells are involved [13]. The question remains whether cells of the recipient react on cells of the donor heart or vice versa. Multiple researchers have studied the mechanism of CAV, and the results were often contradictory. For example, one study revealed that donor dendritic cells (DCs) transmigrate through host secondary lymphoid organs, thereby promoting T-lymphocytes of the recipient, which may promote graft rejection [14]. However, others propose that allo-recognition of donor major histocompatibility complexes (MHC) by recipient immune cells leads to graft rejection [15]. The same is true for the development of fibrosis; are recipient-derived endothelial progenitor cells or endothelial-mesenchymal transition of donor cells responsible for the progressive lesion formation [16, 17]? The immune response could be the initial trigger for fibrosis; however, other mechanisms of fibrosis may be involved as well. In this overview, recipient and donor sides of the stories (immune response and fibrosis) are highlighted to obtain better insights in the pathogenesis of CAV.

## Cardiac Allograft Vasculopathy: Immune Response

### Recipient-Derived Immune Response

**Fig. 2:**
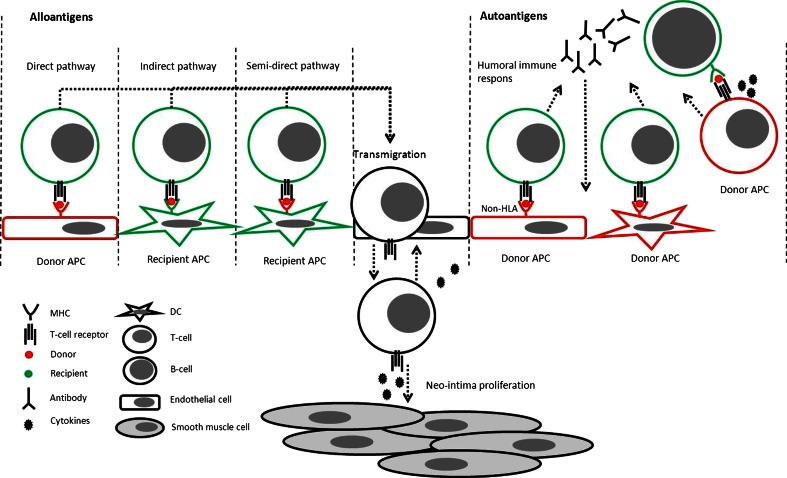
Pathways in recipient-derived immune response. Primary event is the recognition of allo-antigens by T-lymphocytes via one of the indicated pathways. The direct pathway is activated by the recognition of MHC complexes with a foreign HLA-antigen (*red*) presented by donor APCs (*red*). The indirect pathway is activated when T-lymphocytes recognize processed allo-antigens presented by recipient APCs (*green*). The semi-direct pathway is activated when T-lymphocytes recognize allo-antigens presented on donor MHC on recipient APCs. In addition, non-HLA antigens might be involved, which are bound by “auto-antibodies”. This will lead to complement activation and T-lymphocyte activation. Activation of B-cells can be initiated by donor DCs and donor T-lymphocytes. All of the indicated pathways lead to activation of T-lymphocytes, which start to secrete pro-inflammatory cytokines such as IFN-γ. The secretion of IFN-γ recruits more immune cells, such as NK-cells and macrophages, and acts on SMCs. The proliferation of SMCs will ultimately result in proliferation of the intima and occlusion of the artery, which are the characteristics of CAV

According to multiple research groups, the onset of CAV is caused by an immune response of the recipient against the donor [3, 5, 9, 18]. The hypothesis is that after the heart is transplanted, both cellular and humoral immune responses of the recipient are generated against the graft [3]. The immune response of the recipient can be triggered via a (1) direct, (2) an indirect, or a (3) semi-direct pathway (Fig. 2) [18–21]. Although all three pathways can be involved, the semi-direct and direct pathways are less well described in the process of CAV.

(1) In the direct pathway, recipient T-lymphocytes are activated after recognition of allogeneic MHCs (with a foreign antigen) of donor antigen presenting cells (APCs) [18].

(2) The indirect pathway is activated by allo-recognition of processed foreign antigens by APCs of the recipient itself [19]. The recognition of donor antigens on recipient APCs leads to the activation and proliferation of T-lymphocytes. (3) The semi-direct pathway, a new pathway which may be involved, is activated by recipient APCs, presenting donor MHC molecules on their surface [20]. The theory is that recipient APCs acquire donor MHC via cell-cell interaction (intercellular exchange) with donor cells or via the uptake of donor-derived exosomes [21]. The subsequent presentation of donor antigens by donor MHC molecules on recipient APCs will mount a host T-lymphocyte response, leading to the development of chronic rejection.

In all three pathways, the activation of T-lymphocytes will lead to secretion of cytokines such as interleukin-2 (IL-2) and interferon-γ (IFN-γ) [22]. Cytotoxic T-lymphocytes, B-lymphocytes, and macrophages are activated by these cytokines. In addition, endothelial cells are activated and start expressing vascular cell adhesion molecules, which leads to the recruitment of more immune cells [7]. The pro-inflammatory cytokines also enhance the proliferation of smooth muscle cells (SMCs) [15]. Activated B-lymphocytes begin to secrete donor-specific HLA antibodies. These antibodies are important mediators in the development of CAV [23, 24]. They are able to bind the allo-antigens to enable complement factor binding, leading to the activation of the complement system. Furthermore, immune cells, such as macrophages, can bind the donor specific antibodies, which activate antibody-mediated lysis [18]. All of the indicated pathways will ultimately result in vascular injury, ischemia, and damage to the allograft [9].

In addition, there is also evidence that “autoimmunity” plays an important role in the development of CAV [25]. For example, in lung transplant recipients, chronic allograft rejection developed even in the absence of human leukocyte antigen (HLA) antibodies [26]. An explanation for this phenomenon might be the presence of antibodies against non-HLA antigens [25], but the question remains which non-HLA antigens are involved. Recent studies showed that “auto-antibodies” against cardiac myosin and vimentin can be detected after heart transplantation [27, 28]. These “auto-antibodies” are probably induced via antigen mimicry between the donor MHC peptides and auto-antigen peptides of the recipient [28]. T-lymphocytes, which are activated by an indirect allo-immune response, are able to induce chronic rejection by recognition of these auto-antigens [29]. It has been shown in mouse models that induction of tolerance to cardiac myosin leads to a decrease in chronic rejection and an increase in long-term survival after heart transplantation [30]. Therefore, reactivity of the T-lymphocytes toward auto-antigens is likely involved in the development of CAV [28].

Furthermore, the development of anti MHC-class-1-chain-related-A (MICA) antibodies may play a role in the development of CAV [23, 25]. Normally, these antigens are expressed in fibroblasts, monocytes, and endothelial cells [24]. In CAV, there is an increase in MICA expression on endothelial cells [31]. In addition, allo-antibodies against MICA are detected, which actively induce an immune response and cause damage to the endothelium [23].

Cytomegalovirus (CMV) infection has also been known to affect transplantation outcome and CAV. Systemic replication of CMV is associated with increased risk of rejection of the graft and the development of CAV [32]. The theory is that CMV infection induces inflammatory responses of the recipient, thereby contributing to vascular damage and accelerating the pathogenesis of CAV [33].

The trigger of these responses (direct, indirect, and semi-direct pathways, “autoimmunity” and CMV infection) ultimately leads to the proliferation of smooth muscle cells (SMCs), accumulation of extracellular matrix and hyperplasia of the intima of the vessel wall (Fig. 2) [15, 22, 23].

### Donor-Derived Immune Response

Next to the recipient-derived immune response, there is also evidence that donor factors are involved in the immune-pathogenesis of chronic rejection and CAV [34, 35]. Donor factors contributing to CAV include the status of the donor heart and donor-derived cells transplanted during the procedure. The brain death status of the donor greatly influences CAV development [36, 37]. The release of catecholamines during brain death induces endothelial injury leading to cytokine release and MHC up-regulation on donor endothelium [38]. This pathway is mainly investigated in kidney transplant models [37, 39]. However, endothelial dysfunction is generally accepted as one of the strongest predictors of CAV [40]. This process in the donor heart accelerates the early allo-immune response leading to CAV initiation.

Donor-derived cells also play an important role in the pathogenesis of CAV. The current theory is that remaining donor cells within the transplanted heart are able to actively induce an immune response of recipient immune cells [41, 42]. It has been shown that donor-derived immune cells are able to migrate to lymph nodes of the recipient and locally present allo-antigens [43]. Heart-derived donor dendritic cells (DCs) can already be found three hours after transplantation in secondary lymphoid organs and are not as short-lived in recipients as previously thought [41]. A rodent animal study demonstrated that donor DCs can be found in T-lymphocyte areas of the host spleen and hepatic lymphnodes [35]. Hereby, cluster formation of donor DCs and host T-lymphocytes was initiated that activated T-lymphocyte proliferation [35]. These results suggest a donor-derived immune response, initiated by donor DCs.

In addition to donor DCs, the functional activity of donor CD4 T-lymphocytes was studied. In a mouse model, the development of autoimmune reactions after heart transplantation and the contribution to CAV was analyzed [42]. Donor CD4 T-lymphocyte allo-recognition of MHC-II on recipient B-lymphocytes enhanced the production of auto-antibodies, thereby contributing to the development of CAV [42]. When donor CD4 T-lymphocytes were depleted, a significant decrease in both antibody and complement deposition was observed in the allograft [42]. Furthermore, transplant studies showed a mixture (chimerism) of donor and recipient leucocytes, including T-lymphocytes, in heart transplant recipients [44]. However, to what extent donor T-lymphocytes are contributing to CAV after heart transplantation is still unknown.

The expression of donor programmed death-ligand 1 (PD-L1) is also involved in the development of CAV [45]. This ligand plays an important role in the regulation of an allo-immune response by regulating activation of CD4 and CD8 T-lymphocytes [46]. Donor deficiency of PD-L1 accelerates allograft rejection and the development of CAV compared to PD-L1 deficient recipients [45]. Deficiency of donor PD-L1 leads to the secretion of IFN-γ and proliferation of allo-reactive T-lymphocytes of the recipient, thereby promoting a recipient allo-immune response [46]. These findings show that PD-L1 expression on cardiac tissue or leukocytes of the donor is critical in the regulation of an allograft immune response in heart transplant recipients [45, 46].

In addition, it has been shown that donor-derived selectins play an important role in the development of CAV [34]. Selectins are involved in adhesion of leukocytes to the endothelium of the vessel wall [34]. Donor-derived E and P-selectin, located on the endothelium of the graft, interact with L-selectin on recipient-derived leukocytes, thereby enhancing the attraction of immune cells [34]. In rats, there is a significant correlation between the amount of P-selectin expression and intimal thickening of the vessel wall [47]. Corresponding results were found in human recipients with a lung allograft [48]. Furthermore, an increased long-term graft survival with minimal vasculopathy was seen in recipients lacking donor-expressed selectins. This indicates the importance of donor-derived selectins in the development of CAV [49].

According to this accumulating evidence, there is a donor-derived immune response causing the development of allograft vasculopathy and donor cells are involved in regulating the allo-immune response of the recipient. Since this is a relatively new insight, more focus on these aspects is needed to reveal the exact mechanism and to define all of the donor and recipient cells involved.

### Differences in Immune Response in Gender Mismatch Transplantations

Interesting differences in transplantation outcome have been reported between males receiving a female heart or females receiving a male heart, the so called donor-recipient gender mismatch transplantations [50]. Donor-recipient gender mismatch has been shown to influence the early pathogenesis of CAV [51]. At the vascular level, male recipients with a female allograft developed significantly higher amounts of intimal thickening within one year of transplantation [52]. Females receiving a male allograft only developed non-severe thickening of the intima [52]. The combination of male recipients receiving a female heart has been correlated with worse outcomes at several levels besides CAV [53]. Which factors are involved is still under investigation, but there is evidence that smaller heart size, shear stress, and loss of the estrogen-protected environment of the female heart are important factors [1, 52]. Additionally, these factors contribute to initial endothelial damage of the coronary arteries, thereby initiating CAV development. Furthermore, the vasculature of the female heart is thought to be immunologically more susceptible compared to male hearts [52]: a possible explanation is that the vasculature of the female heart expresses more HLA and non-HLA endothelial antigens than their male counterparts [54], which leads to triggering of the male immune system and thereby an earlier development of CAV [52].

At the organ level, contrasting studies showed higher incidents of rejection of female recipients receiving a male heart [55]. These high rejection rates might be explained by greater immuno-competence of the female by developing HLA antibodies against H-Y antigens, presented by cells of the male heart [55]. Presentation of these antigens can lead to an immune response followed by the formation of allogeneic antibodies [25]. The allogeneic immune response against H-Y antigens can lead to graft destruction and ultimately results in rejection of the male heart [56].

Based on these findings, donor-recipient gender mismatch in heart transplantation is followed by dual outcomes. Transplantation of male hearts into females is characterized by higher rejection rates, but in the end, a higher long-term survival [55]. Transplantation of female hearts into males is characterized by an earlier development of CAV [55]. However, some of the studies were limited by the small numbers of gender mismatch transplantations available [51].

## Cardiac Allograft Vasculopathy: Fibrosis

The before-mentioned immune reactions could be followed by fibrosis, which plays an important role in the progressive thickening of the neo-intima and subsequently in the development of CAV [57]. In some recipients, the neo-intima of the coronary arteries almost completely exists of fibrotic tissue [58]. It is known that some inflammatory cells of the recipient, such as T-lymphocytes and macrophages, are involved in the initiation of the fibrotic process [57]. The secretion of cytokines like IFN-γ and transforming growth factor-β (TGF-β) by T-lymphocytes leads to the activation of macrophages and fibroblasts respectively [25, 59]. It has been shown that especially recipient-derived macrophages type 2 (M2) are increased in the neo-intima of CAV arteries [60, 61]. These macrophages are involved in tissue remodeling and matrix deposition and play an important role in the development of fibrotic lesions [60, 62]. They are known to infiltrate the allograft and produce growth factors, such as TGF-β, which increases neo-intimal proliferation [61]. However, more cell types are involved. Next to identified circulating cells of the recipient, there is also evidence of the involvement of donor-derived cells [13, 63].

### Recipient-Derived Circulating Cells

**Fig. 3:**
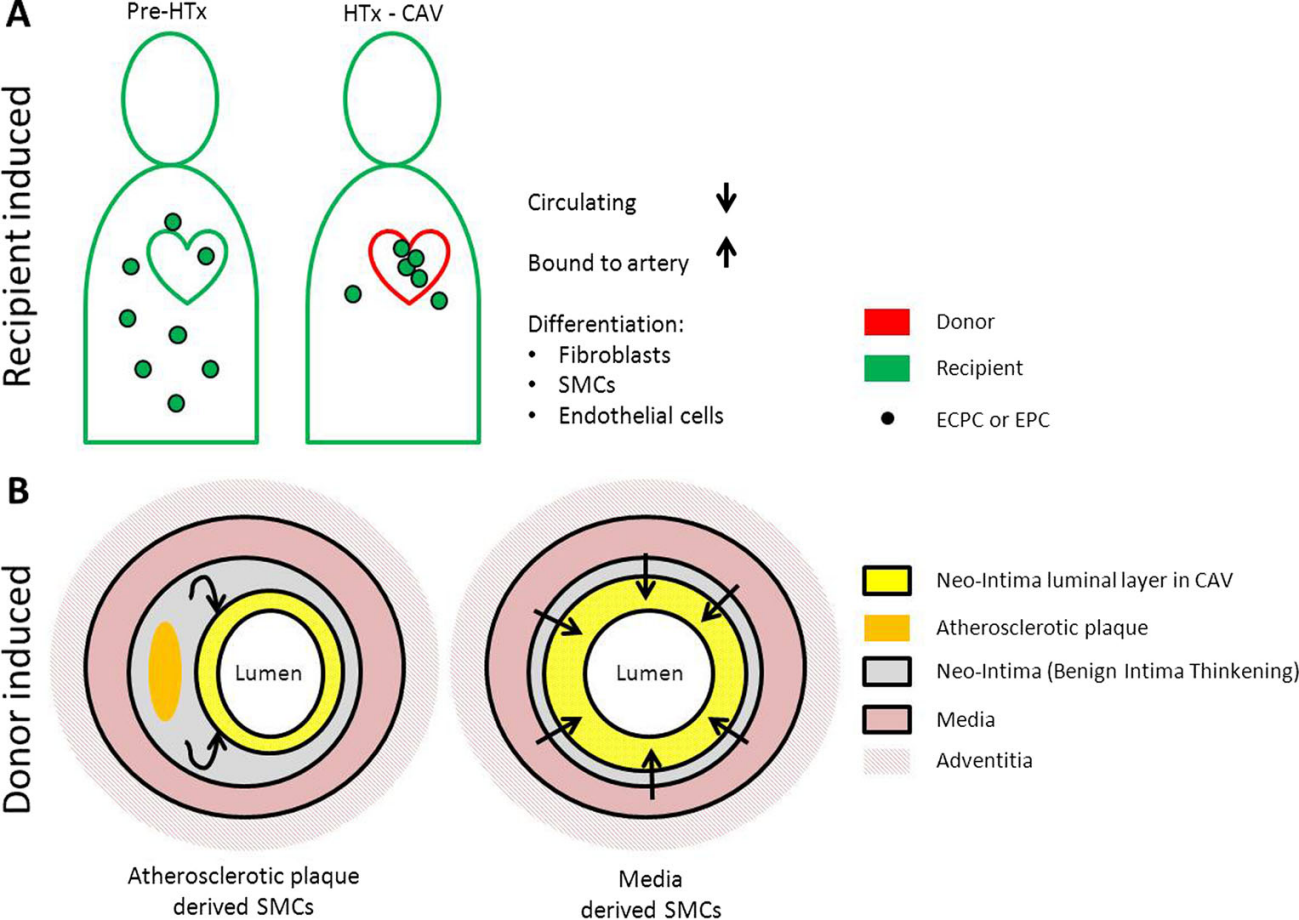
Role of recipient and donor-derived cells in concentric narrowing and fibrosis of the coronary artery. **a** Recipient-derived circulating cells, such as EPCs and ECPCs, contribute to concentric narrowing and fibrosis of the coronary arteries. Increased accumulation of circulating cells bound to the vessel wall induces differentiation of these cells toward fibroblasts, SMCs, and endothelial cells, which enhances concentric narrowing of the coronary arteries. **b**. Donor-derived cells, such as SMCs, are migrating from atherosclerotic lesions or the media layer toward the neo-intima. Accumulation of donor-derived SMCs and production of ECM will lead to the expansion of the neo-intima resulting in narrowing and fibrosis of the coronary artery

There is evidence emerging for the role of recipient endothelial progenitor cells (EPCs) in CAV [29]. EPCs (CD133^+^CD34^+^Flk1 [VEGF-R1]^+^ in bone marrow, CD31 [PECAM-1]^+^CD146^+^vWF^+^NOS^+^ in circulation), are bone marrow-derived cells, which have endothelial regenerative properties [64]. Healthy endothelium of the vessel wall normally undergoes degeneration and regeneration [65]. By an imbalance in these processes, endothelial dysfunction occurs that can lead to injury of the vessel wall [65]. EPCs are able to adhere to the sides of injury and promote healing and repair [66]. Increased numbers of circulating EPCs have been shown to prevent cardiovascular diseases and to reduce neo-intimal hyperplasia in men [67]. However, upon heart transplantation, the protective role of EPCs changes and EPCs of the recipient may participate in the pathogenesis of CAV (Fig. 3a) [16, 65, 68]. Circulating EPCs attach to the vessel wall and start to proliferate as a result of a persistent allograft immune response [68]. The EPCs become uncontrolled, thereby contributing to chronic allograft rejection via accumulation of endothelial cells and SMCs, which in turn leads to occlusive narrowing of the coronary vessels [65]. Upon culturing mononuclear cells from blood, fewer colonies of circulating EPCs were found in heart transplantation recipients with vasculopathy during chronic rejection, compared to transplantation recipients without evidence of vasculopathy [16]. Interestingly, there was also an increase of attached recipient EPCs in the coronary arties of the donor heart, where eventually CAV developed [16]. The hypothesis is that excessive numbers of recipient EPCs differentiate into endothelial cells and SMCs, and that an overload of these cells leads to hyperplasia of the neo-intima and fibrosis [16, 68]. The number of circulating EPCs becomes depleted and thereby the protective effects, as mentioned before, are lost [16].

In addition to EPCs, there is also evidence for a role of recipient-derived extra-cardiac progenitor cells [69]. These extra-cardiac progenitor cells are thought to be derived from mesenchymal precursor cells and migrate toward the allograft where they differentiate into multiple cell lineages [70]. Engraftment of recipient-derived cells in the allograft, such as cardiac progenitor cells, resulted in chimerism of the transplanted heart, which can be beneficial by repopulating the niches of rejected donor cells of the graft [66, 71, 72] However, chimerism of the transplanted heart by extra-cardiac progenitor cells has been shown to be linked to CAV and intimal fibrosis [70]. A possible explanation is that, during cell death of donor cells, an immune response is locally triggered leading to vascular damage of the coronary arteries [64]. In response to tissue injury, recipient-derived mesenchymal precursor cells are attracted and migrate toward the allograft where they differentiate into fibroblasts [73]. In rats suffering from chronic allograft rejection, it has been shown that more than 65 % of fibroblasts in the allograft are of recipient origin [73]. When these fibroblast are activated upon inflammation, they start to proliferate and produce extracellular matrix (ECM) [74]. Ongoing inflammation in chronic rejection leads to a continuous fibrogenic environment, which ultimately leads to fibrosis of the neo-intima in CAV [57, 71, 73, 74].

Although chimerism of the transplanted heart provides evidence for the involvement of recipient-derived circulating cells, the contribution of these cells in the pathogenesis of fibrosis in human CAV is still conflicting and remains to be resolved.

### Donor-Derived Cells

Besides the contribution of recipient-derived cells, there is also evidence that donor cells are involved in the formation of fibrosis [13, 64] It is thought that especially donor-derived SMCs produce extracellular matrix and contribute to the formation of fibrotic lesions in the neo-intima [65]. In CAV, most of the cells in the neo-intima of coronary vessels express markers of SMCs [75]. In human CAV, the majority of these cells are derived from the graft and not from the host [13]. It is not known where these SMCs are originating from. It is possible that resident intimal SMCs expand in number upon inflammation or that they derive from the media and migrate toward the intima to sites with vascular damage (Fig. 3b) [9]. There is also evidence that these donor-derived SMCs originate from endothelial mesenchymal transitions of donor cells [17]. However, the exact role of these donor-derived SMCs needs to be clarified.

Endothelial-to-mesenchymal transition (Endo-MT) and epithelial-to-mesenchymal transition (EMT) have shown to be potential contributors to neo-intima formation [76–78]. Endo-MT is the trans-differentiation of endothelial cells into mesenchymal cells, such as SMCs and fibroblasts, whereas EMT represents the trans-differentiation of epithelial cells into mesenchymal cells [17, 76]. This process normally occurs during certain stages of embryonic development of the heart under influence of TGF-β signaling and is implicated in fibrosis formation [76, 79]. For example, biopsies of human kidney transplants with allograft vasculopathy showed a loss of epithelial markers and an increase in mesenchymal markers [80]. The same trend was observed in studies with cardiac fibrosis, where endo-MT significantly contributed to the development of fibrosis in chronic cardiac disease [81]. It is thought that both endothelial and epicardial-derived cells of the donor, located on the transplanted heart, use this mechanism to differentiate into SMCs and myofibroblasts, thereby contributing to the development of neo-intima fibrosis in CAV [76, 81–83]. Although this hypothesis is gaining attention, it still needs to be unraveled where these donor-derived cells come from.

### Donor-Derived Atherosclerotic Plaques

Next to individual donor-derived cells, it has been suggested that atherosclerotic plaques in coronary vessels of the donor, pre-existing in the transplanted heart, influence the outcome of CAV in the recipient [84]. The atherosclerotic lesions make the intima and the endothelium of the donor coronary arteries more vulnerable for the development of fibrotic lesions during CAV [12]. In these arteries, the fibrotic process and proliferation of immune cells and SMCs was already ongoing in the plaque and could further develop in the transplanted heart, thereby causing neo-intima formation (Fig. 3b). It is also possible that these atherosclerotic lesions develop after transplantation, but there seems to be a correlation between pre-existing atherosclerotic lesions and a more fibrotic CAV outcome [8].

In conclusion, it appears that not only inflammatory cells of the donor are involved in the pathogenesis of CAV, but also donor-derived cells, such as endothelial cells, epicardial cells, and smooth muscle cells. In addition, presence of atherosclerotic plaques might be correlated to fibrotic lesions in CAV. These findings provide new insights in a possible role for the donor in the development of neo-intima fibrosis in CAV; however, this is still under debate.

## Interventions

Activation of the immune system of the recipient, for example by contact of donor- and recipient cells, should be avoided. Ideally, when transplanting a solid organ, there is no transfer of donor immune cells to trigger an immune response. In the future, this might be achieved by, for instance, ex vivo perfusion of the donor organ, clearing all immune cells from the graft [85]. When ex vivo heart perfusion would be applicable in future clinical practice, therapies necessary for that specific donor organ could be applied to prolong graft survival as experimentally tested in lung transplantation [86, 87]. These therapies could target pathways involved in endothelial damage, cell death, ischemia reperfusion-injury, and many other processes related to CAV. For each donor organ, “personalized medicine” could be applied with the specific factor to restore the damaged organ and get better long-term outcomes after transplantation.

Multiple prophylactic approaches have been used to prevent the development of CAV [88]. Modification of risk strategies may slow the progression of CAV, including the prevention of CMV infection and endothelial damage caused by the immune response in general [89]. Environmental infection of CMV after transplantation can activate immune cells of donor and recipient [33]. However, most likely, this would not have an effect on the long-term fibrotic response.

Currently used therapeutic interventions, such as statins, angiotensin-converting enzyme (ACE) inhibitors and immunosuppressive medications are not always successful in the treatment of CAV [88]. These therapies are not specifically developed for CAV, but are developed for cardiovascular diseases in general. Statins for example, did show anti-inflammatory properties by significantly reducing acute cellular rejection episodes, however, were never proven to prevent CAV [90, 91]. Therefore, it is most likely that they do not specifically inhibit the immune- and fibrotic processes in CAV. The most advanced therapy is the use of proliferation inhibitors, a new class of immunosuppressants, such as everolimus and sirolimus [92]. These are able to reduce intimal thickening in CAV by inhibiting cell proliferation [93]. In CAV, proliferation of immune cells and SMCs is described, which would suggest that these proliferation inhibitors are effective as long proliferation of these cells would occur. Therefore, timing of these therapies is very important, since early after transplantation, they disrupt fibroblast function and therefore wound healing and late after transplantation, they don’t show any effects [94, 95]. These proliferation inhibitors showed promising results in preliminary studies, however, remain not implicated in most clinical centers [96].

## Conclusions

CAV is the leading cause of death after heart transplantation, although the pathogenesis is not fully resolved [1]. Until recently, CAV has been considered to be induced by either a recipient-derived immune response or a donor-derived immune response [5]. However, more evidence points to the possibility of a dual action of donor and recipient. By comparing both sides of the story, more knowledge about the pathogenesis of CAV can be obtained (see Table 1 for summary of presented findings).

**Table 1:** Summary based on recent findings of interacting cells derived from both donor and recipient in the pathogenesis of CAV

Cardiac allograft vasculopathy: immune response	
Recipient-derived immune response	Donor-derived immune response
Alloimmune response	
[1] Direct pathway	Donor DCs
[2] Indirect pathway	Donor CD4+ T-lymphocytes
[3] Semi-direct pathway alloantibodies MICA	Donor-derived selectin expression
	Donor-derived PD-L1 expression
Auto-immune response “auto-antibodies”	
Differences in gender mismatch transplantations Male recipients: higher rejection rate, higher long-term survival Female recipients: earlier development CAV	
Cardiac allograft vasculopathy: fibrosis	
Recipient-derived circulating cells	Donor-derived cells
Circulating cells T-lymphocytes macrophages type-2 fibroblasts	Donor cells Smooth muscle cells myofibroblasts
Progenitor cells endothelial progenitor cells extra-cardiac progenitor cells	Mesenchymal transitions endothelial—mesenchymal cells epicardial—mesenchymal cells
	Presence of donor-derived atherosclerotic plaques

In conclusion, CAV is a complex disease with an unrevealed pathogenesis. Presumably, CAV is not induced only by the donor or recipient. Based on current research, it is clear that both donor and recipient cells are involved [9, 42, 43]. It appears the immune system of the recipient is the most important player in the development of CAV, since immune activation of the recipient initiates allograft immune responses, which ultimately leads to vascular damage [10]. Even without the interference of donor-derived immune cells, CAV can probably develop. Donor immune cells, derived from the transplanted heart, are able to enhance the immune response of the recipient, but it seems that they are not able to induce CAV independently. Most likely, there is some kind of synergic interaction between recipient and donor cells, which accelerates the pathogenesis of CAV. More research is needed to completely identify the dual interaction of both donor and recipient cells. Some studies were using only animal experiments or experimental CAV; therefore, it would be interesting to investigate whether the outcomes of animal studies are consistent in human CAV samples. The outcomes based on previous experimental models should in the future be extrapolated to the human transplant recipient to elucidate the potential enhancing role of donor cells in the pathogenesis of CAV.

## Abbreviations

αSMA: Alpha smooth muscle actin
ACE: Angiotensin-converting enzyme
APC: Antigen-presenting cell
CAV: Cardiac allograft vasculopathy
CMV: Cytomegalovirus
DC: Dendritic cell
ECM: Extracellular matrix
ECPC: Extra cardiac progenitor cells
EMT: Epithelial-to-mesenchymal transition
Endo-MT: Endothelial-to-mesenchymal transition
EPC: Endothelial progenitor cells
H-CAV: Histopathological CAV
HLA: Human leukocyte antigen
IFN-γ: Interferon-γ
IL: Interleukin
MHC: Major histocompatibility complex
MICA: MHC class 1 chain-related A
PD-L1: Programmed death-ligand1
SMC: Smooth muscle cell
TGF- β: Transforming growth factor-β
